# High-Quality Metal–Organic Framework ZIF-8 Membrane Supported on Electrodeposited ZnO/2-methylimidazole Nanocomposite: Efficient Adsorbent for the Enrichment of Acidic Drugs

**DOI:** 10.1038/srep39778

**Published:** 2017-01-04

**Authors:** Mian Wu, Huili Ye, Faqiong Zhao, Baizhao Zeng

**Affiliations:** 1 Key Laboratory of Analytical Chemistry for Biology and Medicine (Ministry of Education), College of Chemistry and Molecular Sciences, Wuhan University, Wuhan 430072, Hubei Province, P. R. China

## Abstract

Metal–organic framework (MOF) membranes have received increasing attention as adsorbents, yet the defects in most membrane structures greatly thwart their capacity performance. In this work, we fabricated a novel ZnO/2-methylimidazole nanocomposite with multiple morphology by electrochemical method. The nanocomposite provided sufficient and strong anchorages for the zeolitic imidazolate frameworks-8 (ZIF-8) membrane. Thus, a crack-free and uniform MOF membrane with high performance was successfully obtained. In this case, 2-methylimidazole was believed to react with ZnO to form uniform ZIF nuclei, which induced and guided the growth of ZIF-8 membrane. The as-prepared ZIF-8 membrane had large surface area and good thermal stability. As expected, it displayed high adsorption capacity for acidic drugs (e.g., ibuprofen, ketoprofen and acetylsalicylic acid) as they could interact through hydrophobic, hydrogen bonding and π-π stacking interaction. Accordingly, by coupling with gas chromatography the ZIF-8 membrane was successfully applied to the real-time dynamic monitoring of ibuprofen in patient’s urine.

Metal–organic frameworks (MOFs) are a class of crystalline inorganic–organic hybrid materials with well-defined porous structures. Compared with conventional inorganic porous materials, MOFs possess better porosity and larger specific surface areas, especially, their pore sizes and surface function can be easily tuned upon the selection of different metal ions and organic bridging ligands^1^,^2^,^3^. These properties make them ideal candidates for applications in catalysis^4^, sensors^5^ and sorption-related fields^6^,^7^,^8^,^9^,^10^,^11^,^12^,^13^. Up to now, MOFs have been widely exploited for gas separation^6^, gas storage^7^,^8^, and adsorption of volatile organic compounds^9^,^10^. Moreover, the adsorption and removal of pharmaceuticals (e.g. furosemide and sulfasalazine)^11^, dyes^12^, alkylaromatics and phenols^13^,^14^, and sulfur compounds^15^,^16^,^17^ from liquid phase by using MOFs have also been reported. MOFs, particularly, the zeolitic imidazolate frameworks (ZIFs) are superb candidate materials for preparing membranes^18^,^19^. However, the fabrication of high-quality and practical MOF membranes for adsorption remains a big challenge due to the poor nucleation, adhesion and intergrowth of MOF crystals^19^.

Various methods have been reported for the fabrication of MOF membranes, including *in situ* growth without^20^,^21^,^22^ or with substrate modification^23^,^24^, seeded growth^25^,^26^,^27^, along with others^28^,^29^,^30^,^31^. It is often difficult to prepare MOF membranes by the *in situ* growth method due to MOFs’ poor heterogeneous nucleation sites on substrates. When using the seeded growth method, it is important to prepare high quality MOF seeds and to immobilize them on support surface, as demonstrated by Lai who synthesized a ZIF-8 membrane on alumina discs by a seeding approach^25^. Gascon *et al*. used this approach to grow a Cu_3_(BTC)_2_ membrane on α-alumina^26^. However, they were unable to obtain a uniform, highly ordered, smooth membrane free of cracks or intercrystal gaps.

A dual metal source method has also been used to prepare MOF membranes^32^,^33^,^34^, in which the formation mechanism of MOF is tactfully explored. Moreover, this method does not need the procedures of seed preparation and deposition. ZnO, as an excellent metal source for MOF membrane growth, has been investigated^35^,^36^,^37^. However, if ZIF-8 is grown on ZnO directly, the ligands will react with the Zn^2+^ in solution and a large amount of homogeneous nucleation sites will occur because ZnO cannot provide adequate heterogeneous nucleation sites for continuous MOF membranes. To deal with this problem, activation process comes into being, which is handled in a methanol solution containing 2-methylimidazole (Hmim) ligand^36^,^37^. Nevertheless, the activation process is time and reagent consuming, and the activation reagent is usually not friendly to the environment. Recently, Li *et al*.^38^ fabricated defect-free MOF membranes on PVDF hollow fiber by using ZnO array as a buffering layer, in which no activation procedure was needed. Furthermore, MOF layer was strongly adhered to the hollow fiber, and the membranes possessed excellent gas separation performance.

Inspired by Li’s idea, we prepared novel ZnO/2-methylimidazole (ZnO/Hmim) nanocomposites with multiple morphologies on pencil bars by electrochemical deposition. To study the effect of the concentration of organic ligand Hmim on the morphology of the nanocomposite, a series of ZnO/Hmim nanocomposites were synthesized by changing Hmim concentration. Using the obtained nanocomposites as intermediate layer, we successfully fabricated defect-free and uniform ZIF-8 membranes, in which the activation procedure could be completely omitted (Fig. 1). It should be addressed that our synthesis strategy here is different from others. During this synthetic process, organic ligand Hmim was believed to react with ZnO to create active sites on the surface of pencil bar and made it possible for the nucleation and growth of a crack-free ZIF-8 membrane without activation procedure. The obtained ZIF-8 membrane had large surface area and good thermal stability. Excitingly, it exhibited higher adsorption capacity for acidic drugs (e.g., ibuprofen, ketoprofen and acetylsalicylic acid) compared with commercial polydimethylsiloxane/divinylbenzene (PDMS/DVB) adsorbent, as they could interact through hydrophobic, hydrogen bonding and π-π stacking interaction. Therefore, the ZIF-8 membrane is a promising material for adsorption and separation.

## Results and Discussion

### Morphology of the ZnO/Hmim nanocomposites

The alkali-etched pencil bar presented a rougher tree bark-like structure with striped appearance compared with the untreated one (see Supplementary Figure S2). This indicated that sodium hydroxide reacted with silicate in the pencil bar and made it form porous structure, thus the surface roughness of the pencil bar increased, which was benificial for the attachment of ZnO layer onto its surface.

A 10 μm thick nanoporous ZnO layer was electrodeposited on the pencil bar as shown in Fig. 2. The ZnO nanosheets interconnected and tightly attached onto the pencil bar surface. They uniformly covered over the entire surface of pencil bar to serve as an intermediate support layer for ZIF-8 membrane. In this layer the pore diameter was roughly 400 ± 50 nm (see Fig. 2a).

The doping of Hmim is crucial for ZIF-8 deposition. The organic ligand Hmim could be adsorbed and react with ZnO to generate nuclei, which promotes the deposition and growth of ZIF-8 membrane. Experiment results showed that ZIF-8 deposition was poor on both bare pencil bar and ZnO layer coated pencil bar without Hmim doping (see Supplementary Figure S3).

With Hmim doping, spheroidal particles evenly dispersed on the pore cavity, and their diameters were about 300 nm (Fig. 3a). These microparticles were believed to be nuclei formed by the reaction of Hmim and ZnO, which provided adequate anchorages for ZIF-8 membrane. The FTIR spectra revealed that Hmim was integrated in the composite (note that the peaks marked with dotted line originated from Hmim) (Supplementary Figure S4), which was beneficial for the synthesis of ZIF-8 membrane and allow for the omission of the activation step. X-ray diffraction of the composite also displayed the diffraction lines belonging to the pencil bar (i.e. graphite) and ZnO from the nanosheet layer (Fig. 3f).

As the concentration of Hmim was an important factor affecting the morphology of the ZnO/Hmim, 25, 50, 75 and 100 mM Hmim were used (Fig. 3a–e). The alkaline of the solution increased with the increase of Hmim concentration, while this phenomenon was not distinct until the solution reached saturation at 100 mM. At 25 mM, the spheroidal microparticles dispersed on the pore cavity, but not filled. After Hmim concentration increased to 50 mM, small protuberances formed on the surface of microparticles. The layer thickness was around 10 μm. And then at 75 mM, these protuberances grew into cone with diameter of about 200 nm, almost filled the pore cavity. When Hmim concentration reached 100 mM, the structure and size of conical particle stayed unchanged. The thickness of these ZnO/Hmim layers was similar to that of ZnO layer, indicating that the reaction of Hmim and ZnO was conducted in the pore cavity of nanoporous ZnO. The XRD patterns (Supplementary Figure S5) and FTIR spectra (Supplementary Figure S6) of these ZnO/Hmim composites were similar.

### ZIF-8 membrane preparation

As reported in previous studies^36^,^37^, the activation of ZnO was found to be vital for ZIF-8 membrane formation. But here, because the ZnO nanosheets were doped with Hmim to generate nuclei, uniform ZIF-8 layer could be prepared without a ZnO activation procedure. As shown in Fig. 4, a 20 μm thick well-intergrown and impacted layer of ZIF-8 membrane was grown on the surface of ZnO/Hmim nanocomposite. On the membrane surface, no cracks, pinholes, or other defects were observed, indicating that a high-quality membrane had been made. It was noticeable that the crystals were tightly anchored to the substrate, indicating a strong adhesion to the pencil bar. Furthermore, the ZnO layer was not obvious in the SEM images. This may be because the metal oxide, as the metal ion source, participated in the reaction^39^.

The XRD diffractograms confirmed that the material was crystalline and contained the same phase as ZIF-8 (Supplementary Figure S7). For ZIF-8 membrane, the ZnO phase had almost completely disappeared, which was in agreement with the SEM images. To check the effect of the ZnO/Hmim morphology on the preparation of ZIF-8 membrane, we grew ZIF-8 layer on the pencil bar with different ZnO/Hmim nanocomposites, but the obtained ZIF-8 membranes were similar.

The prepared ZIF-8 membrane gave a BET surface area of 1830 m^2^/g with a pore volume of 0.98 cm^3^/g (see Fig. 5A). The TGA data revealed that the ZIF-8 membrane was stable up to 350 °C, indicating that the thermostability of the ZIF-8 was sufficient for solid-phase microextraction (SPME) application (Fig. 5B).

### Adsorption properties of ZIF-8 for acidic drugs

Studies regarding the adsorption of acidic drugs have been extensively reported^40^,^41^,^42^,^43^,^44^,^45^,^46^. However, only limited investigations used ZIF-8 adsorbent to adsorb acidic drugs. ZIF-8 membrane was selected for the adsorption studies of acidic drugs because of its remarkable thermal stability and flexible adsorption manner.

The time-dependent adsorption capacity was obtained to discuss the adsorption kinetics of ibuprofen on ZIF-8 (Supplementary Figure S8a). The adsorption capacity of ibuprofen increased significantly in the first 2 h, and reached equilibrium gradually. The equilibrium time for low concentration ibuprofen was somewhat longer than that for high concentration. According to Fick’s law, the diffusion of low concentration of analytes across the ZIF-8 membrane slowed down^47^. Moreover, the adsorption capacity significantly increased as the initial concentration of ibuprofen increased, indicating the favorable adsorption at high concentrations. The equilibrium adsorption capacity increased from 0.009 to 0.05 mg/g as initial concentration varied from 10 to 250 μg/L. The adsorption kinetics of ibuprofen on PDMS/DVB was also studied for comparison (Supplementary Figure S8b). The equilibrium time was longer than that for ZIF-8. On the other hand, the equilibrium adsorption capacity was lower. The reason was that the PDMS/DVB coating was much thicker, which hindered the diffusion of analytes across the coating. Even so, PDMS/DVB coating couldn’t provide enough adsorption sites.

The adsorption data well fit the Langmuir model and can be expressed linearly as:


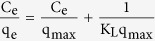


where *q*_e_ is the adsorption capacity at equilibrium, its unit is mg/g, *q*_max_ is the saturation capacity of the adsorbent, *C*_e_ is the ibuprofen concentration in solution at equilibrium, and *K*_L_ is the Langmuir constant and it’s an important parameter characterizing the adsorption capacity of solid surface. The plots of *C*_e_/*q*_e_ against *C*_e_, constructed according to Fig. 6A, gave straight lines (Fig. 6B), and the values of *q*_max_ and *K*_L_ were determined from the slopes and intercepts of the plots (Table 1).

As displayed in Fig. 6 and Table 1 the Langmuir model fit the experimental data particularly well, and the *R*^2^ value was greater than 0.982. The ZIF-8 membrane presented a saturation capacity of 0.051 mg/g for ibuprofen, much higher than PDMS/DVB. This was because ibuprofen could be strongly adsorbed on the surface of ZIF-8 via hydrophobic effect, hydrogen bonding interaction and π-π stacking interaction originating from Hmim molecules. Also, the larger surface area of ZIF-8 provided more active adsorption sites than PDMS/DVB.

The adsorption isotherms for ketoprofen and acetylsalicylate acid on ZIF-8 and PDMS/DVB were also investigated. Ketoprofen and acetylsalicylate acid showed saturation capacity of 0.039 mg/g and 0.045 mg/g acetylsalicylate acid on the ZIF-8 respectively (Supplementary Figure S9 and Tables S1 and S2).

### Selectivity of ZIF-8

The selectivity of ZIF-8 membrane was evaluated by testing a series of organic compounds, including volatile aliphatic compounds and benzene series. At the same time, PDMS/DVB was selected for comparison. The comparison was conducted by calculating the adsorption amounts of analytes, and the results were shown in Figure S10. Generally, PDMS/DVB coating shows little selectivity for compounds with different functional groups. However, ZIF-8 membrane exhibited higher adsorption amounts for all the analytes studied than PDMS/DVB coating. Furthermore, enhanced selectivity was obtained for benzene series, owing to the π–π affinity between the framework Hmim and phenyl ring in these compounds. Therefore, ZIF-8 membrane had good selectivity for compounds containing phenyl ring, including the acidic drugs in this work.

### Determination of ibuprofen in urine samples

The resulting ZIF-8 membrane was applied to the analysis of urine sample spiked with ibuprofen, wide linear range (0.025–250 μg/L) and low limit of detection (LOD, 13.5 ng/L) were obtained (Supplementary Table S3). Compared with the previous methods (Supplementary Table S4), this method presented higher sensitivity and/or lower LODs. This was related to the high adsorption capacity of ZIF-8 membrane. The repeatability and reproducibility were also explored, for five ZIF-8 coated pencil bars prepared independently by the same procedure, the average relative standard deviation (RSD) of the chromatographic peak signal was 6.9% (n = 5), while five successive measurements of 10 μg/L ibuprofen using a ZIF-8 coated pencil bar gave a RSD of 4.3% (n = 5). In addition, the adsorption performance of the ZIF-8 membrane was monitored during its use, and no obvious loss was observed after it had been used for more than 100 times. Moreover, the crystal structure of ZIF-8 was well-kept according to the XRD characterization shown in Figure S11.

Then the ZIF-8 membrane was applied to monitor the concentration of ibuprofen in urine from a patient with fever colds. The sampling was conducted every one hour after he took the medicine (from 9:00 am to 6:00 pm). During this 10-h sampling period, extraction was conducted intermittently, with each spot lasting for 0.5 h. Since GC running time was also 0.5 h, extraction with only one ZIF-8 coated pencil bar for each sampling period was conducted in this experiment. The body temperature was monitored using a clinical thermometer every hour. The concentrations of ibuprofen at different sampling time were shown in Fig. 7. The peak concentration was detected at 1:00 am, three hours after taking the medicine. This indicated that the absorption of ibuprofen in human body was quick. Besides the spot concentration data, the time weighted average (TWA) concentration can be determined by dividing the sum value of all time weight concentrations by the total sampling time. Each time weight concentration can be calculated by multiplying the spot concentrations by its time weighting. The obtained TWA concentration of ibuprofen over the 10-h sampling was 34.6 ng/L (shown as “TWA_Ave” in Fig. 7).

## Conclusions

In summary, a defect-free ZIF-8 membrane with high performance was successfully fabricated by using a ZnO/Hmim nanocomposite as support layer. Our experiments demonstrated that ZnO/Hmim nanocomposite with diverse morphologies could be deposited on pencil bars by electrochemical method. It served as intermediate support layer for growth of high-quality ZIF-8 membrane without any activation procedure. The as-prepared ZIF-8 membrane presented excellent adsorption capacity for acidic drugs (saturation capacities of 0.051, 0.039 and 0.045 mg/g for ibuprofen, ketoprofen and acetylsalicylic acid, respectively) and it could be applied to the monitor of ibuprofen in urine.

## Experimental Section

### Materials and chemicals

The pencil bars (6.0 cm × 0.3 mm O.D.), purchased from Sinopharm Chemical Reagent Company (China), were cut into length of 2 cm and cleaned (see the Supporting Information). The chemicals and reagents used for the ZIF-8 membrane preparation included zinc sulfate (≥99%), potassium chloride (≥99.5%), boric acid (≥99.5%) for the deposition of ZnO nanosheet layer, and zinc chloride (≥98%), sodium formate dihydrate (≥99.5%), methanol anhydrous (≥99.5%) for the growth of ZIF-8 membrane. Hmim (99%), acidic drugs (ibuprofen (98%), ketoprofen (99%) and acetylsalicylic acid (99%)) were purchased from Sigma-Aldrich Chemical. Co. Ltd. Aliphatic compounds (octanol, nonanol, decanol, undecanol, dodecanol, nonanal, decanal, 3-octanone), benzene series (toluene, ethylbenzene, chlorobenzene, o-xylene and p-xylene) were supplied by Sinopharm Chemical Reagent Company. All chemicals were used without further purifications. Ultrapure water was used throughout this work. Stock solutions of acidic drugs at 1 mg/mL were prepared, and all of them were refrigerated for storage. Working solutions were prepared by step-by-step dilution with methanol just before use.

### Preparation of ZIF-8 membranes

**Figure 1:**
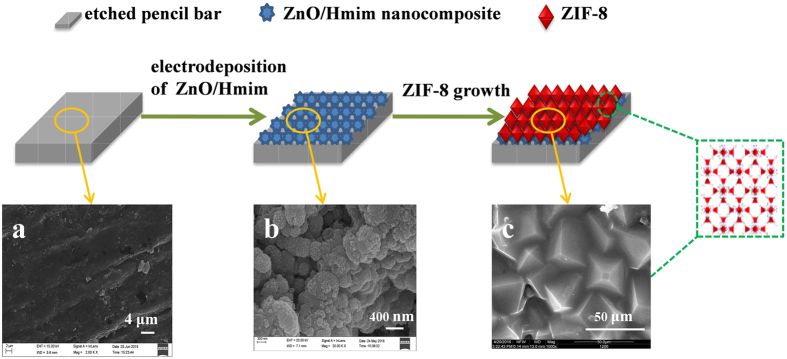
Schematic illustration of the two-step fabrication process of ZIF-8 membrane supported on ZnO/Hmim nanocomposite and SEM images of etched pencil bar (**a**), after electrodeposition of ZnO/Hmim (**b**), and after growth of ZIF-8 (**c**).

**Figure 2:**
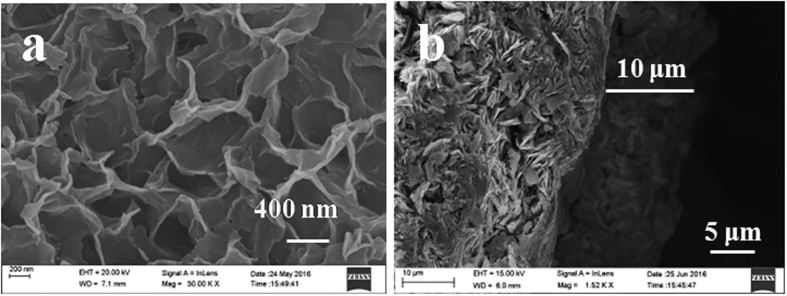
SEM images of ZnO nanosheets on the surface of pencil bar. (**a**) top view, (**b**) cross section.

**Figure 3:**
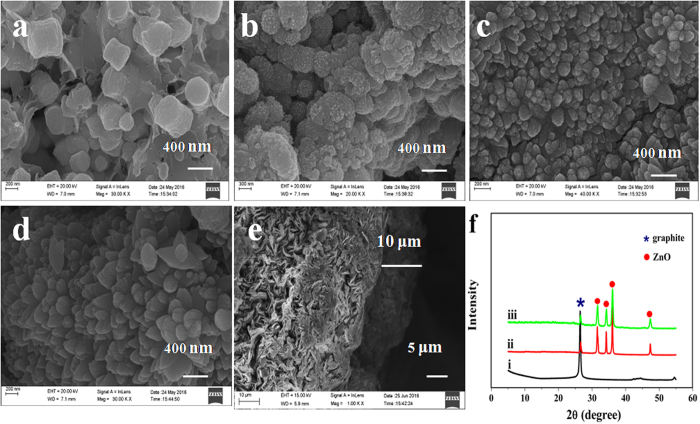
SEM images of ZnO/Hmim composites (**a**–**e**) on the surface of pencil bar. (**a**–**d**) top view, (**e**) cross section; Hmim concentration: (**a**) 25 mM (pH = 5.52), (**b**) 50 mM (pH = 5.89), (**c**) 75 mM (pH = 6.23), (**d**) 100 mM (pH = 6.29), (**e**) 50 mM. (**f**) X-ray diffractograms of the bare pencil bar support (i), ZnO (ii) and ZnO/Hmim composite (iii).

**Figure 4:**
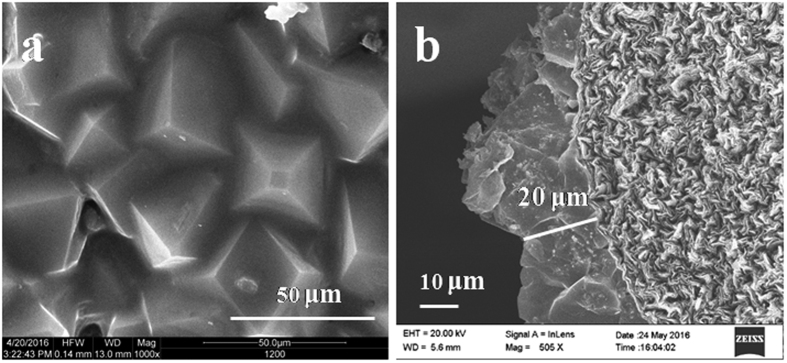
SEM images of ZIF-8 membrane grown on ZnO/Hmim composite. (**a**) top view, (**b**) cross section.

**Figure 5:**
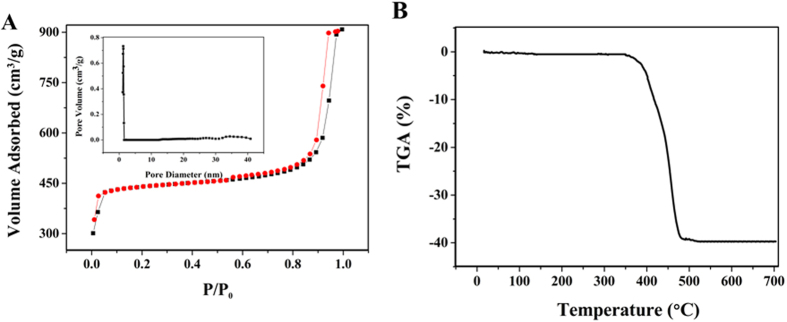
(**A**) N_2_ adsorption–desorption isotherms and the pore size distribution of the as-synthesized ZIF-8 (inset). (**B**) TGA curve of the as-synthesized ZIF-8.

**Figure 6:**
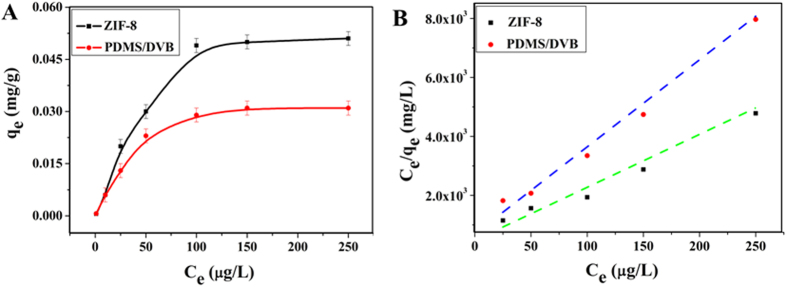
(**A**) The adsorption isotherms of ibuprofen on ZIF-8 and PDMS/DVB at 50 °C. (**B**) The adsorption isotherms of ibuprofen on ZIF-8 and PDMS/DVB in their linearized format and fit by the Langmuir model.

**Figure 7:**
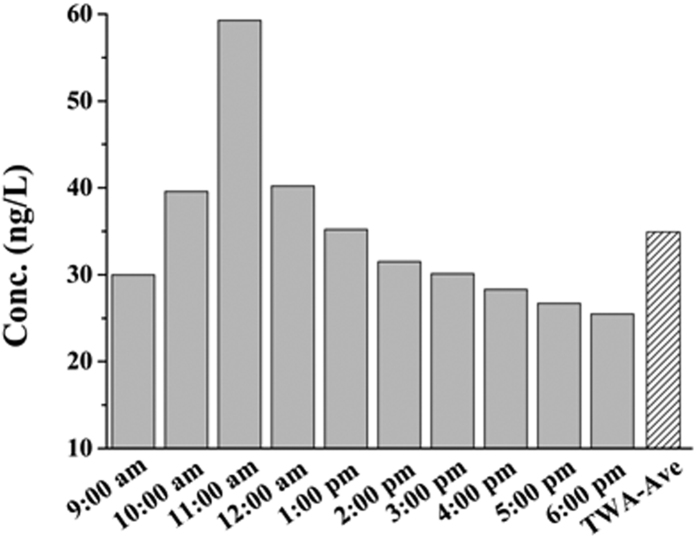
Monitoring of ibuprofen concentration in a patient’s urine. Adsorption temperature, 50 °C; adsorption time, 30 min; desorption temperature, 300 °C; desorption time, 5 min.

The preparation procedure of ZIF-8 membrane involved (1) electrochemical deposition of ZnO/Hmim nanocomposite on the pencil bar and (2) synthesis of ZIF-8 membrane as illustrated in Fig. 1.

The ZnO/Hmim particles were directly electrodeposited on the sodium hydroxide etched pencil bar in 10 mL aqueous solution containing zinc sulfate (30 mg/mL), potassium chloride (200 mg/mL), boric acid (25 mg/mL) and Hmim (25–100 mM). The deposition potential was −1.2 V (vs SCE), the solution temperature was 30 °C, and the deposition time was 15 min. The obtained ZnO/Hmim particles coated pencil bar was rinsed with ultrapure water to remove the unreacted reactants, and dried at 60–80 °C overnight. For comparing study, ZnO nanosheets were electrodeposited on the pencil bar under the same conditions but without Hmim.

The ZIF-8 membrane was prepared according to literature with minor modifications^38^. Briefly, a solid mixture of 0.181 g zinc chloride, 0.164 g Hmim and 0.09 g sodium formate was dissolved in 10 mL anhydrous methanol by ultrasonic treatment. ZnO/Hmim-coated and ZnO-coated pencil bars were placed horizontally in a Tefion-lined stainless steel autoclave, which was filled with the synthesis solution, and heated (at 85 °C) for 24 h. After the solvothermal synthesis, the ZIF-8 layer coated pencil bars were thoroughly washed with methanol to remove any unreacted residues, and dried in oven at 60 °C over night.

### Adsorption experiments

The adsorption behaviors of three acidic drugs on ZIF-8 membrane were studied at a controlled temperature. The ZIF-8 coated pencil bar was assembled into a homemade SPME device (see Supplementary Figure S1) and then conditioned in the gas chromatography (GC) injector at 300 °C under nitrogen for 1 h prior to use. The pH of the working aqueous solution, containing 0.36 g/mL NaCl, ibuprofen, ketoprofen and acetylsalicylic acid at a desired concentration, was pre-adjusted with NaOH and HCl. To initiate the experiments, 10 mL working aqueous solution was transferred to a 15 mL vial capped with polytetrafluoroethylene coated septum. Adsorption was performed at 50 °C under magnetic agitation at 500 rpm. The needle of the SPME device was pierced through the septum, and the ZIF-8 coated pencil bar was exposed to the headspace for a controlled time. After adsorption, the needle was removed from the vial and immediately transferred to the GC injection port for thermal desorption at 300 °C for 5 min, followed by GC-FID determination for subsequent analysis. For a kinetic study, the adsorption was carried out by varying the acidic drug concentrations (ranging from 1 to 250 μg/L) at constant temperature (50 °C), and the chromatographic signals were collected at different time intervals for the determination of adsorbed analytes. When the adsorption amount no longer increased with increasing time, indicating equilibrium adsorption was reached. Thus isothermal adsorption curves were worked out.

The adsorption of ibuprofen from urine was described in the Supporting Information.

### Characterization

The scanning electron microscopy (SEM) images were obtained using an LEO 1530 field emission SEM (Carl Zeiss NTS GmbH, Germany). X-ray diffraction data (XRD) were recorded with a Bruke D8 diffractometer (Germany) using Cu Kα radiation (40 kV, 40 mA) with a Ni filter in the range of 5–55°. FT-IR spectra were recorded with a Nexus-670 Fourier transform infrared spectrometer (Nicolet, USA). Prior to FT-IR analysis, samples were dried under vacuum at 95 °C for 24 hours, and analytical grade KBr was dried 24 hours in an oven at 120 °C. Sample and KBr were finely ground using an agate mortar and pestle, then pressed into a pellet for analysis. Nitrogen adsorption–desorption measurements were conducted with a Micromeritics ASAP-2020 M analyzer at 77 K. The sample was degassed at 150 °C for 6 h in vacuum before the tests. The Brunauer, Emmet, and Teller (BET) method was used to calculate the surface area of the adsorbents. Thermogravimetric analysis (TGA) of ZIF-8 was performed on a Netzsch-209 thermal gravimetric analyzer (Bavaria, Germany) from room temperature to 700 °C in flowing N_2_ at heating rate of 10 °C/min.

## Additional Information

**How to cite this article:** Wu, M. *et al*. High-Quality Metal–Organic Framework ZIF-8 Membrane Supported on Electrodeposited ZnO/2-methylimidazole Nanocomposite: Efficient Adsorbent for the Enrichment of Acidic Drugs. *Sci. Rep.* **7**, 39778; doi: 10.1038/srep39778 (2017).

**Publisher's note:** Springer Nature remains neutral with regard to jurisdictional claims in published maps and institutional affiliations.

## Supplementary Material

Supplementary Information

## Figures and Tables

**Table 1:** Langmuir model parameters for ibuprofen adsorption with PDMS/DVB and ZIF-8.

Material	q_max_ (mg/g)	K_L_ (L/μg)	R^2^
PDMS/DVB	0.031	0.046	0.982
ZIF-8	0.051	0.065	0.990
