# Supplementary material for: High-Quality Metal–Organic Framework ZIF-8 Membrane Supported on Electrodeposited ZnO/2-methylimidazole Nanocomposite: Efficient Adsorbent for the Enrichment of Acidic Drugs
Source: Sci Rep. 2017 Jan 4;7:39778. doi: 10.1038/srep39778 (PMC5209695; doi:10.1038/srep39778)
Supplement: Supplementary Information [file srep39778-s1.doc]

**Supporting information for**

**High-Quality Metal−Organic Framework ZIF‑8 Membrane Supported on Electrodeposited ZnO/2-methylimidazole Nanocomposite: Efficient** **Adsorbent for** **the Enrichment of Acidic Drugs**

Mian Wu, Huili Ye, Faqiong Zhao, Baizhao Zeng[[1]](#footnote-2)*

Key Laboratory of Analytical Chemistry for Biology and Medicine (Ministry of Education), College of Chemistry and Molecular Sciences, Wuhan University, Wuhan 430072, Hubei Province, P. R. China

Corresponding author: Baizhao Zeng

Tel. : 86-27-68752701

Fax : 86-27-68754067

**Supplement of experimental section**

**The treatment of pencil bar**

The pencil bar was dipped into a 1.0 M NaOH solution (70 °C) for 30 min to enhance surface roughness. Subsequently the pretreated pencil bar was washed repeatedly with distilled water and ethanol with the aid of sonication, followed by drying in an oven at 100 °C for 5 h.

**Instrumentation**

A CHI 617A electrochemical workstation (CH Instrument Corp., Shanghai, China) was employed for preparing SPME fibers, which was in junction with a conventional three-electrode system, including a pencil bar (2 cm × 0.3 mm O.D.) as working electrode, a Pt counter electrode (2.5 cm × 0.1 cm O.D.) and a saturated calomel electrode (SCE) as reference electrode. The heating and stirring of the samples were carried out by using a model S10-3 heater-magnetic stirrer (Shanghai). The analysis of the acidic drugs was performed on a GC-2010 gas chromatography system (Shimadzu Corporation, Japan) fitted with a split/splitless injection chamber, HP-5 column (30 m, 0.25 mm i.d., 0.25 μm film thickness) (USA) and a flame ionization detector (FID). A GC solution chromatographic workstation program (Shimadzu Corporation) was used to process chromatographic data. Ultrapure nitrogen was used as carrier gas at a constant flow rate of 1 mL/min. Hydrogen and air ﬂow rates were maintained at 40 mL/min and 400 mL/min, respectively. The temperatures of capillary splitless injector and detector were 300 °C. The column temperature was initially set at 50 °C for 3 min, ramped at 10 °C/min to 140 °C, ramped at 5 °C/min to 200 °C, ramped at 10 °C/min to 220 °C, and held for 4 min, and the total run time was about 30 min. The SPME device was laboratory-made. Commercial 65 μm polydimethylsiloxane/divinylbenzene (PDMS/DVB) SPME ﬁber was purchased from Supelco (Bellefonte, PA).

**SPME of ibuprofen from urine sample**

The urine sample was provided by a healthy volunteer. It (10 mL) was transferred into 15 mL vials with polytetrafluoroethylene silicon septum, and was spiked with ibuprofen standard solution to 0.025 - 250 μg/L. Then NaCl was added to saturation, and the solution pH was maintained at 3. The SPME of ibuprofen was performed at 50 °C for 30 min under magnetic agitation at 500 rpm. Subsequently, the fiber was placed in the GC injection port for desorption of 5 min at 300 °C.

**Supporting figures**

**
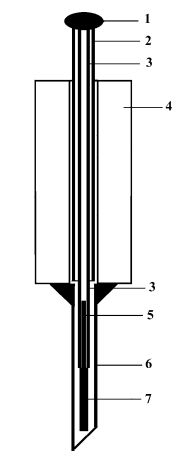
**

**Figure S1** Sketch view of the vertical section of the developed SPME device: 1, handle grip; 2, push-pull rod (stainless steel tube, 100 mm length × 1.1 mm o.d. × 0.8 mm i.d.); 3, stainless steel capillary (150 mm length × 0.30 mm o.d. × 0.10 mm i.d.); 4, main body of the microsyringe (calibrated glass tube); 5, etched part of the pencil bar; 6, needle of the microsyringe (50 mm length × 0.6 mm o.d. × 0.4 mm i.d.); 7, ZIF-8 coated pencil part.


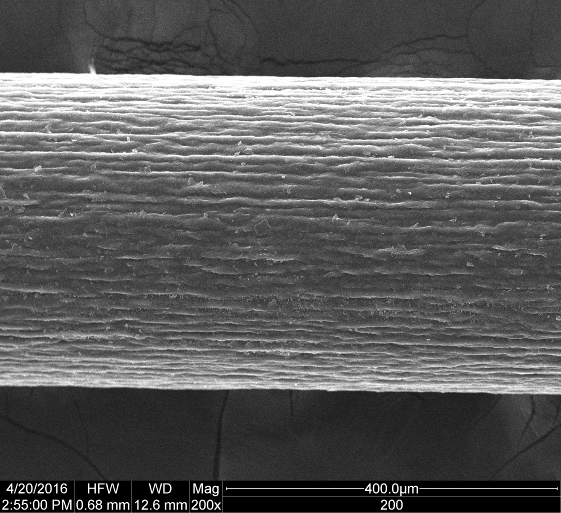


**a**

**200 μm μm**


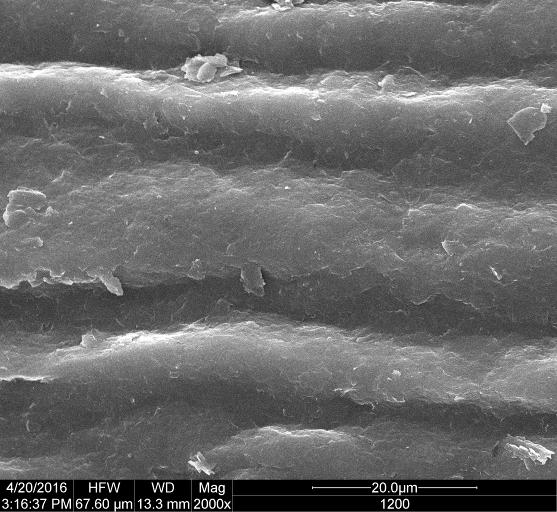


**b**

**20 μm μm**


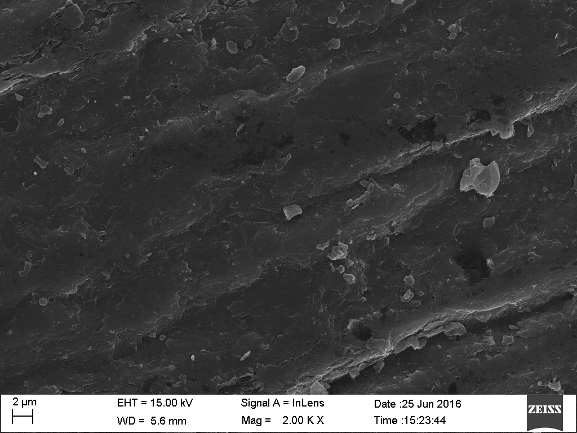


**d**

**4 μm**


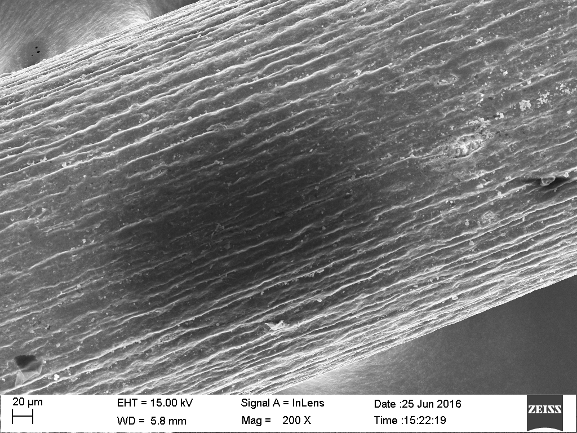


**40 μm**

**c**

**Figure S2** SEM images of pencil bar before (a,b) and after treating with NaOH solution (c,d). (a,c) low magnification, (b,d) high magnification.


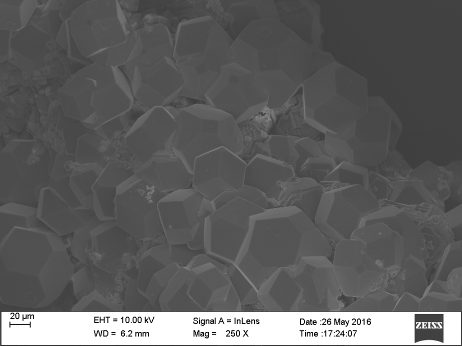


**a**

**40 μm μm**


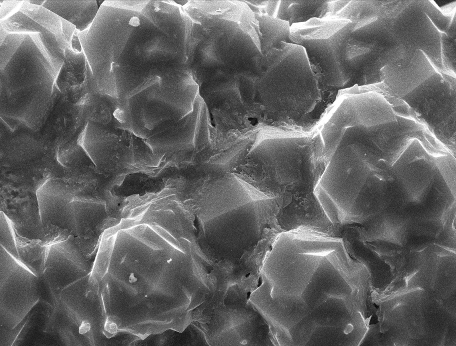


**b**

**25 μm μm**


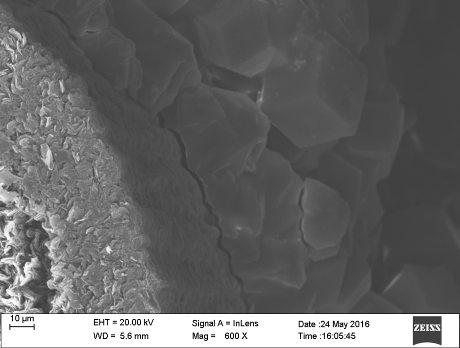


**c**

**20 μm**


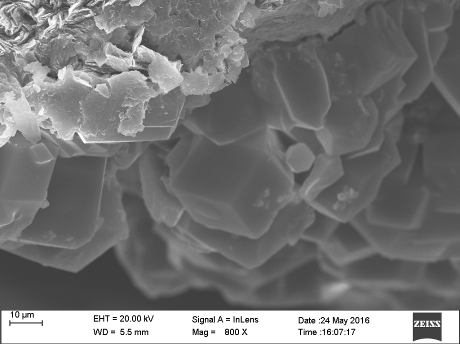


**d**

**10 μm**

**μm**

**substrate**

**ZIF-8 crystal**

**ZIF-8 crystal**

**substrate**

**Figure S3** SEM images of ZIF-8 crystal grown on bare pencil bar (a,c) and ZnO layer modified pencil bar (b,d). (a, b) top view, (c, d) cross section.

**Figure S4** FTIR of the ZnO and ZnO/Hmim.

**Figure S5** XRD patterns of ZnO/Hmim. Hmim concentration: (a) 25 mM (pH = 5.52), (b) 50 mM (pH = 5.89), (c) 75 mM (pH = 6.23), (d) 100 mM (pH = 6.29).

**Figure S6** FTIR of ZnO/Hmim. Hmim concentration: (a) 25 mM (pH = 5.52), (b) 50 mM (pH = 5.89), (c) 75 mM (pH = 6.23), (d) 100 mM (pH = 6.29).

**Figure S7** XRD pattern of ZIF-8 membrane.

**Figure S8** Effect of contact time on the adsorption of ibuprofen (at different initial concentrations) on ZIF-8 (a) and PDMS/DVB (b) at 50 °C. q_t_: adsorption capacity (mg/g).

**Figure S9** (A) Ketoprofen adsorption isotherms for ZIF-8 and PDMS/DVB at 50 °C. (B) Ketoprofen adsorption isotherms for ZIF-8 and PDMS/DVB in their linearized format and fit by the Langmuir model. (C) Acetylsalicylic acid adsorption isotherms for ZIF-8 and PDMS/DVB at 50 °C. (D) Acetylsalicylic acid adsorption isotherms for ZIF-8 and PDMS/DVB in their linearized format and fit by the Langmuir model.

**Figure S10** Adsorption amounts obtained with the ZIF-8 membrane and PDMS/DVB for aliphatic compounds (A) and benzene series (B). The concentration of each analyte in aqueous solution was 50 μg/L; adsorption time, 30 min; adsorption temperature, 50 °C. The error bars represented the standard deviations (n = 3).

**Figure S11** XRD patterns of ZIF-8 membrane before and after adsorbing ibuprofen.

**Supporting tables**

**Table S1** Langmuir model parameters for ketoprofen adsorption with PDMS/DVB and ZIF-8.

| Material | q_max_ (mg/g) | K_L_ (L/μg) | R^2^ |
| --- | --- | --- | --- |
| PDMS/DVB | 0.034 | 0.041 | 0.996 |
| ZIF-8 | 0.039 | 0.056 | 0.986 |

**Table S2 Langmuir model parameters for acetylsalicylic acid adsorption with PDMS/DVB and ZIF-8.**

| Material | q_max_ (mg/g) | K_L_ (L/μg) | R^2^ |
| --- | --- | --- | --- |
| PDMS/DVB | 0.037 | 0.039 | 0.990 |
| ZIF-8 | 0.045 | 0.052 | 0.970 |

**Table S3 Analytical parameters for ibuprofen measured with ZIF-8 membrane.**

| Analyte | Regression equation | Linear range  (μg/L) | R | LOD  (ng/L) | RSD (%) | |
| --- | --- | --- | --- | --- | --- | --- |
|  |  |  |  |  | One bar  (n=5) | Bar to bar  (n=5) |
| ibuprofen | y = 1780 x + 8972 | 0.025−250 | 0.9987 | 13.5 | 4.3 | 6.9 |

**Table S4** Comparison of several methods used for the determination of ibuprofen.

| Detection method | Linear range (μg/L) | LODs  (μg/L) | Ref. |
| --- | --- | --- | --- |
| µ-SPE-HPLC | 1-250 | 0.08 | [40] |
| IT-SPME-LC | - | 2 | [41] |
| LLE-GC-MS | - | 1.207 | [43] |
| SPME-HPLC | 0.3-650 | 0.1 | [44] |
| SSPE-LC-UV | 0.2-750 | 9 | [45] |
| SPME-LC | - | 0.9 | [46] |
| SPME-GC-FID | 0.025-250 | 0.0135 | This work |

µ-SPE: micro-solid-phase extraction

HPLC: high-performance liquid chromatography

IT-SPME: in-tube solid phase microextraction

LLE: liquid-liquid extraction

MS: mass spectrometry

SSPE: supramolecular solid-phase extraction

1. ^^ Corresponding author. Tel: 86-27-68752701, Fax: 86-27-68754067.

   E-mail address: bzzeng@whu.edu.cn [↑](#footnote-ref-2)
